# Paradoxical Effect of Chloroquine Treatment in Enhancing Chikungunya Virus Infection

**DOI:** 10.3390/v10050268

**Published:** 2018-05-17

**Authors:** Pierre Roques, Simon-Djamel Thiberville, Laurence Dupuis-Maguiraga, Fok-Moon Lum, Karine Labadie, Frédéric Martinon, Gabriel Gras, Pierre Lebon, Lisa F. P. Ng, Xavier de Lamballerie, Roger Le Grand

**Affiliations:** 1 Immunology of Viral Infections and Autoimmune Diseases (IMVA), IDMIT Department, Institut de Biologie François-Jacob (IBJF), Univ. Paris-Sud–INSERM U1184, CEA, 92265 Fontenay-aux-Roses, France; laurdupuis@gmail.com (L.D.-M.); karine.labadie-bretheau@cea.fr or klabadie@genoscope.cns.fr (K.L.); frederic.martinon@cea.fr (F.M.); gabriel.gras@cea.fr (G.G.); roger.le-grand@cea.fr (R.L.G.); 2 IRD, INSERM U1207, EHESP French School of Public Health, UMR190, Aix-Marseille University, 13005 Marseille, France; djamt@yahoo.fr (S.-D.T); xavier.de-lamballerie@univ-amu.fr (X.d.L.); 3 Singapore Immunology Network, Agency for Science, Technology and Research, Biopolis 138648, Singapore; Lum_Fok_Moon@immunol.a-star.edu.sg (F.-M.L.); lisa_ng@immunol.a-star.edu.sg (L.F.P.N.); 4 Service de Virologie, AP-HP, Hôpital Cochin, Paris Descartes University, 75014 Paris, France; pflebon2@wanadoo.fr

**Keywords:** alphavirus, chikungunya, chloroquine, monocyte-macrophage, macaque

## Abstract

Since 2005, Chikungunya virus (CHIKV) re-emerged and caused numerous outbreaks in the world, and finally, was introduced into the Americas in 2013. The lack of CHIKV-specific therapies has led to the use of non-specific drugs. Chloroquine, which is commonly used to treat febrile illnesses in the tropics, has been shown to inhibit CHIKV replication in vitro. To assess the in vivo effect of chloroquine, two complementary studies were performed: (i) a prophylactic study in a non-human primate model (NHP); and (ii) a curative study “CuraChik”, which was performed during the Reunion Island outbreak in 2006 in a human cohort. Clinical, biological, and immunological data were compared between treated and placebo groups. Acute CHIKV infection was exacerbated in NHPs treated with prophylactic administration of chloroquine. These NHPs displayed a higher viremia and slower viral clearance (*p* < 0.003). Magnitude of viremia was correlated to the type I IFN response (Rho = 0.8, *p* < 0.001) and severe lymphopenia (Rho = 0.8, *p* < 0.0001), while treatment led to a delay in both CHIKV-specific cellular and IgM responses (*p* < 0.02 and *p* = 0.04, respectively). In humans, chloroquine treatment did not affect viremia or clinical parameters during the acute stage of the disease (D1 to D14), but affected the levels of C-reactive Protein (CRP), IFNα, IL-6, and MCP1 over time (D1 to D16). Importantly, no positive effect could be detected on prevalence of persistent arthralgia at Day 300. Although inhibitory in vitro, chloroquine as a prophylactic treatment in NHPs enhances CHIKV replication and delays cellular and humoral response. In patients, curative chloroquine treatment during the acute phase decreases the levels of key cytokines, and thus may delay adaptive immune responses, as observed in NHPs, without any suppressive effect on peripheral viral load.

## 1. Introduction

Chikungunya fever (CHIKF) is caused by chikungunya virus (CHIKV), which is an arthropod-borne virus that was first identified in Tanzania in 1952–1953 [1]. Acute infection is usually characterized by severe arthralgia and myalgia, which may persist for months or years after the initial febrile episode (for review, [2]). The introduction of CHIKV into the Americas in December 2013 through the Caribbean islands has led to a resulting 2.3 million suspected cases, with more than 541 associated deaths as of December 2017 (PAHO; http://www.paho.org).

Despite extensive studies, no effective antiviral drug is available for CHIKV prevention or treatment (for review, [3]). Numerous compounds demonstrated anti-CHIKV properties in cell cultures, however failure to evaluate these compounds in suitable animal models has limited their potential use in humans [4]. Current treatments have been mainly palliative using antipyretic, analgesic, and anti-inflammatory drugs [5]. Chloroquine, which is a typical anti-malaria drug, has displayed antiviral properties in vitro [6,7,8,9,10,11]. CHIKV infection in Vero E6 cells was strongly inhibited following chloroquine treatment [12,13]. It was thus shown that chloroquine inhibit the early step of the viral infection in pre-treatment assay by modifying the endosomal pH, but also at some extant budding stage when being used as co-treatment [10,13]. The effectiveness of chloroquine in protecting against CHIKF was assessed in a double-blinded, placebo-controlled randomized trial in Reunion Island “CuraChik” conducted during the Reunion Island outbreak in 2006 [12,14]. Despite its antiviral potential, oral chloroquine treatment for five days in patients with acute chikungunya did not protect against severe disease. Moreover, the first analysis has shown that chronic arthralgia on day 300 post illness onset, was more frequent in patients receiving chloroquine [12,15]. This suggests that chloroquine may have exacerbated the disease and/or suppressed the antiviral immunity, leading to a chronic disease [12].

To understand the reasons behind the failure of chloroquine treatment in CHIKV-infected patients, we performed two complementary studies: (i) a prophylactic chloroquine treatment in a preclinical non-human primate (NHP) model of CHIKV infection [16], and (ii) a retrospective study of immunological parameters in patients that were recruited to the “CuraChik” clinical trial that was conducted during the Reunion Island outbreak in 2006.

## 2. Materials and Methods

### 2.1. Prophylactic Treatment in a Preclinical NHP Model

#### 2.1.1. Animals

Three- to four-year-old male cynomolgus macaques (*Macaca fascicularis*), weighing 3–4 kg, were imported from Mauritius (negative for SIV, STLV, herpes B virus, filoviruses, SRV-1, SRV-2, measles, Dengue virus, and CHIKV) and were housed in a BSL3 facility (Permit Number A 92-032-02), in accordance with Office for Laboratory Animal Welfare (OLAW, Bethesda, MD, USA; #A5826-01) standards. Studies were approved by the regional animal care and use committee in accordance with European directive 63/2010/EU: “CREEA Ile de France Sud”, Fontenay aux Roses, decision #A08-012 dated 7 July 2008. Treatment and sampling procedures caused no suffering. At the end of the study, animals were sedated and euthanized by the intra-venous (i.v.) injection of a lethal dose of pentobarbital.

#### 2.1.2. Viral Stock

The CHIKV strain LR2006-OPY1 was used, as previously described [16]. The in vitro titer was 10^8^ CCID50/mL in BHK21 cells and 1.8 × 10^10^ ± 0.9 vRNA equivalents per mL. In vivo titer was obtained by infection of eight cynomologus macaques with serial dilutions of the virus stock. The 50% animal infectious dose (AID50) was estimated at 7.07 × 10^3^ ± 3.15 vRNA copies [17].

#### 2.1.3. Animal Treatment and Infection

Animals were treated with chloroquine once daily either orally (dose of 7 or 14 mg/kg, 0.83 g/mL in saccharose syrup; 5 mg/mL chloroquine sulfate (Baby Nivaquine^®^, Avantis-Pharma, Antony, France), or subcutaneously (14 mg/kg chloroquine diphosphate (Sigma, Saint Louis, MO, USA); 30 mg/mL in PBS. Subcutaneous treatments were thus 1.6 to 2 mL of a PBS solution. The days with treatment but not bleeding, animals received chloroquine after containment, but not sedation. Animals were sedated with ketamine (10 mg/kg, Rhône-Mérieux, Marcy l’Etoile, France) before handling. Clinical examinations, rectal temperature, and weight measurements were performed 10 min after sedation, before bleeding. Macaques were inoculated i.v. with 100 AID50 in 1 mL PBS, 1 h after the 6th treatment. Plasma chloroquine concentration was determined by high-performance liquid chromatography (HPLC), as described elsewhere [18]. Aspartate transaminase (AST), alanine transaminase (ALT), and Complement-Reactive Protein (CRP) levels were evaluated using Gamme DPC Kit AST/GOT or ALT/GPT (Thermo Electron Corporation/Thermo Fisher Scientific, Saint Herblain, France), following the manufacturer’s instructions. Complete blood count was obtained with a Micro Diff II apparatus (Beckman Coulter, Villepinte, France).

#### 2.1.4. In Vitro Infection of Primary Macrophages and Fibroblast Cells

Human monocytes were isolated from PBMCs with CD14 magnetic beads (Miltenyi Biotec, Bergisch Gladbach, Germany) and were cultured for six days in 24-well plates containing DMEM (^©^Glutamax, Gibco Lifesciences/Thermo Fisher Scientific, Saint Herblain, France), supplemented with 10% fetal calf serum (FCS, (PAA, Les Mureaux, France)), M-CSF and GM-CSF (10 and 1 ng/mL, R&D Systems, Abingdon, UK). Macaque fibroblasts were obtained from tendons after necropsy (macaque negative for CHIKV or Dengue virus infection as assessed by antibodies and PCR evaluation). Briefly, the tendons were minced and plated in 3 cm diameter Petri dishes that were saturated with FCS. Tendon pieces are immerged within DMEM, 20% FCS and explant are cultured at 37 °C, 9% CO2, water saturated atmosphere. After 7 to 10 days, fibroblasts might be seen migrating from the explants that should be discarded (Supplementary Figure S1A). Then, the cells were passaged every three days in DMEM, 10% FCS, and stable up to 13 passages. The resulting macrophages (Day 7 post-isolation, 10^5^ cells/well) or fibroblasts (passage 5, day 23 post-isolation, 4 × 10^5^ cells/well), respectively, were incubated with various concentrations of chloroquine for 24 h, before being infected by incubation with CHIKV (multiplicity of infection (MOI) at 1 or 3.4, respectively) for two hours at 37 °C. They were then thoroughly washed five times and 900 µL of culture medium supplemented by chloroquine at the same concentration was subsequently added.

#### 2.1.5. Plasma Viral RNA Extraction and Quantification

Viral RNA was prepared from 100 µL of cell-free plasma collected in EDTA tubes, and quantified, as previously described [16]. The standard RNA template dilution gave a correlation coefficient of 98–99% over seven orders of magnitude, with a sensitivity of 10^3^ vRNA/mL or 20 copies per sample.

#### 2.1.6. Virion-Based Ig ELISA

Inactivated purified CHIKV (kindly provided by Alere, Brisbane, Australia) was immobilized on 96-well Maxisorp plates (Nunc, Roskilde, Denmark). Wells were blocked by overnight incubation at 4 °C with 0.05% Tween-20 (*v*/*v*), 5% non-fat dried milk (*w*/*v*) in PBS (PBST-milk). Plasma samples were diluted 1:150 to 1:109,000 in PBST-milk and were incubated for one hour at 37 °C in the plates. Horseradish peroxidase (HRP)-conjugated anti-rhesus IgG and anti-human IgM (Southern Biotech, Birmingham, AL, USA) were used to detect macaque IgG and IgM, respectively. Reactions were developed with TMB substrate (3, 3′, 5, 5′–Tetramethylbenzidine, Sigma) and were terminated by adding stop reagent (Sigma). Samples from non-infected NHPs were used as controls. ELISA was performed in duplicate.

#### 2.1.7. Cytokine and ELISPOT Assays

IFNα/β bioassay was performed as previously described [19]. Plasma levels of selected cytokines and growth factors were measured using the Miliplex Non-Human Primate Cytokine Magnetic Bead Panel for 23 soluble markers: GM-CSF, TGFα, G-CSF, IFNγ, IL-2, IL-10, IL-15, sCD40L, IL-17, IL-1Ra, IL-13, IL-1β, IL-4, IL-5, IL-6, IL-8, MIP-1α, MCP-1 (CCL2), TNFα,MIP-1β, IL-12–23(p40),IFNγ,IL-18; or, the Human Cytokine assay Magnetic Bead on 10 soluble factors: Eotaxin, GM-CSF, IFNα2, IL-12(p70), IL-1Ra, IL-6, IL-8, IP-10, MCP-1, TNFα, respectively (Millipore, Darmstadt, Germany), according to the manufacturer’s instruction. Data was acquired with a Bio-Plex Instrument 200 and analyzed with Bio-Plex Manager Software version 6.1 (Bio-Rad, Hercules, CA, USA). IFNγ ELISPOT was performed, as previously described [20], with heat-inactivated CHIKV (45 min, 56 °C twice; 10^7^ virions per well in 100 µL).

### 2.2. Curative Treatment from the CuraChik Clinical Trial

CuraChik was a randomized double blind, placebo-controlled, prospective trial aiming at evaluating the efficacy and the safety of chloroquine as therapeutic treatment of CHIK. The main results of this study are already reported [12,14]. Briefly, this trial was achieved during the Reunion island outbreak in 2006 and included adult patients (18–65 years old, men and women), who presented an acute febrile arthralgia within less than 48 h. Patient inclusion and follow-ups were exclusively carried out by general practitioner (GPs) that was involved in the study.

Blood human samples were taken after obtaining the informed consent from the patients (http://clinicaltrials.gov/ct2/show/NCT00391313, as described [12]) in accordance with the tenets of the Declaration of Helsinki.

Here, we presented only the data from patients confirmed positive by CHIKV RT-PCR on day 1 (D1) and seroconversion of CHIKV-specific antibodies on day 16 (D16), for which serum samples were available for the immunological assays.

After inclusion and a first clinical and biological assessment (biochemistry and viral load using CHIKV RT-PCR at day 1), patients were randomly grouped in a double-blind procedure to either the chloroquine group (600 mg at day 1, 600 mg at days 2 and 3, and 300 mg at days 4 and 5) or the placebo group.

Clinical data from the patients were collected through a daily questionnaire (D1–D14), three consultations with the GPs (D1, D7, and D25), and through two telephone questionnaires (D100 and D300). Biological data were collected from blood samples drawn on D1, D3, D6, and D16, as previously described [15].

#### Biochemical Analysis of Human Samples

C-reactive protein (CRP) and aspartate transaminase/alanine transaminase (AST/ALT) levels were determined with Konelab 20 version 6.0.7 (Thermo Fisher Scientific, Maltham, MA, USA), Konelab “CRP Plus” and AST/GOT and ALT/GPT (DPC^®^T) kits (Thermo Fisher). The coefficient of variation for low CRP values was high (7%), so a quantification limit (QL) of 5 mg/L was used.

Cytokine assays were performed as described above for the NHP assay.

### 2.3. Statistical Analysis

Nonparametric Spearman and Wilcoxon rank tests, Mann and Whitney (M&W), and Chi^2^ tests for non-normal distributions were performed with StatView5.0 (SAS Institute, Cary, NC, USA) or Prism5.0d (GraphPad Software, La Jolla, CA, USA). Anova one-way non-parametric Friedman and Dunn’s correction was done with Prism7.0. Viral production over time was assessed by calculating the area under the curve (AUC) with Prism5.0d.

For the human study analysis, chloroquine and placebo groups were compared at baseline using Fisher’s exact test for qualitative factors, the M&W test for continuous factors, and Spearman nonparametric test for correlation assessment. To assess the impact of chloroquine on the acute disease evolution we performed a generalized linear model with a Generalized Estimating Equation (GEE) approach [21]. GEE analyzes the evolution of the response over time and the influence of the covariates. The correlation matrix has been defined as auto-regressive. Dependant variables were modelled using a linear model or a Poisson distribution depending on the dependent variable. Factors relating to the last clinical assessment (D300) were estimating using a backwards-stepwise multivariate linear regression models (logistic, Gaussian, or Poisson regression, depending on the independent variable, respective percentage, continuous, or count variable). Variables with P-values <0.2 were retained and entered into multivariate analysis. Multivariate analyses were done based on the Akaike information criterion (AIC). When there were several competing models, the fitting model that gives the minimum AIC was considered to be the one with statistically maximum likelihood. All of the statistical analyses were performed with the IBM SPSS statistic version 19 software (IBM Corp, Armonk, NY, USA).

Values of *p* < 0.05 were considered significant.

## 3. Results

### 3.1. Pre-Clinical Studies

#### 3.1.1. Chloroquine Inhibits CHIKV Replication in Monocyte-Derived Macrophages and Fibroblasts

It has been reported that chloroquine inhibits CHIKV infection efficiently in a variety of cells [12,13], albeit to differing extents. Fibroblasts and macrophages are two cellular targets during CHIKV infection, and it has been shown that macrophages play a key role in chikungunya pathogenesis [16,22]. Therefore, we first investigated treatment of primary fibroblasts and macrophages with varying concentrations of chloroquine (1 to 50 µM) for 24 h before infection with CHIKV at a multiplicity of infection (MOI) of 1 or 3.4, respectively, which were shown to induce the same kinetics of viral replication in both cell types.

**Figure 1:**
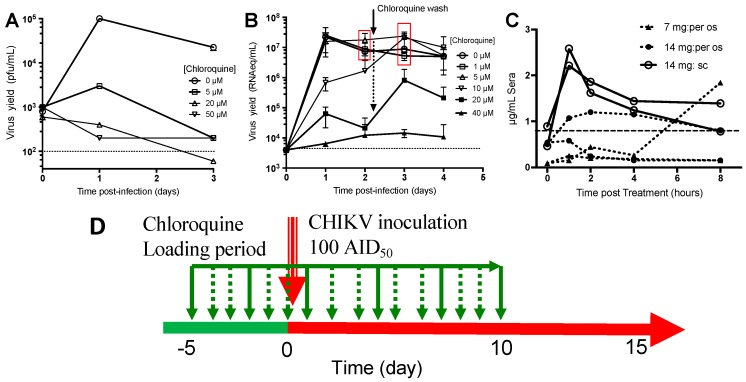
Chloroquine inhibition of Chikungunya virus (CHIKV) replication in cynomolgus macaque cells in vitro and pharmacokinetics in vivo. (**A**) Antiviral activity of chloroquine against the CHIKV infection of macaque primary monocyte-derived macrophages (MDM, quadruplicate; 4–5 × 10^5^ MDM per well) were treated for 24 h with various concentrations of chloroquine, infected with a MOI of 3.4. CHIKV levels in the supernatant were quantified by titration on the BHK-21 sensitive cell line, as previously described; (**B**) Primary Fibroblast cells derived from macaque tendon (quadruplicate; 4 × 10^5^ per well) were treated for 24 h with various concentrations of chloroquine, infected with a multiplicity of infection (MOI) of 1. CHIKV levels in supernatant were quantified by direct RT-PCR in 30µL of the culture supernatant. At day 2 post-infection, all cells pre-treated with less than 10 µM of Chloroquine and exposed to the virus are dying (red square). The remaining chloroquine was washed out at day 2 and a virus rebound is assessed in the 10 and 20 µM chloroquine treated cells that were killed day 3. Cells that were treated with 40 µM chloroquine were protected from infection but abnormal structures and shape were seen (Supplementary Figure S1b). The data shown either in panel A or B are representative of two experiments, dashed lines are lower limit of quantification; (**C**) Two macaques per regimen were treated for five days with chloroquine. On day 5, blood was taken just before the 6th treatment (time 0), for Cmin determination, or at 1, 2, 4, or 8 h after chloroquine administration. Dashed horizontal line is for Cmin obtained in Human and in macaque after five days of treatment with 14 µg/kg/days of chloroquine. Serum chloroquine concentration was determined as described in the Methods section; (**D**) Scheme of the treatment procedure used during in vivo assay. Chloroquine administration was shown in dotted or solid green arrow, CHIKV inoculation in red arrow and period of infection in animal on red horizontal line.

At the highest dose used, chloroquine did not affect the number of macrophages in culture. Interestingly, at day 1 post-infection, chloroquine treatment with 20 and 50 µM decreased viral yield in CHIKV-infected macrophages by more than two logs. In fact, treatment with just 5 µM chloroquine decreased viral yield by approximately 1.5 logs (Figure 1A). At day 3 post-infection, all of the viral yields decreased by more than two logs regardless of the concentrations of chloroquine used, and viral loads were no longer detectable in long-term in vitro treatment [12,13]. On the other hand, fibroblasts were sensitive to chloroquine concentration, as they were killed when treated with 50 µM of chloroquine. CHIKV infection was lethal, and the fibroblasts were killed when treated with 1 and 5 µM of chloroquine (Figure 1B). Thus, in contrast to macrophages, chloroquine at these concentrations were not effectively against CHIKV in fibroblasts. However, chloroquine treatments at 10 µM and 20 µM were effective to supress CHIKV replication, resulting in a decrease in viral yield of at least 1 log at day 2 post-infection. Interestingly, washing out of chloroquine (10 and 20 µM regiments) at day 2 post-infection (Figure 1B, red box), allowed for CHIKV recovery, leading to greater viral replication, and eventually, increased cell death (Figure 1B). It was also observed that CHIKV replication rebounded at day 3 post-infection after washing out the chloroquine at 20 µM chloroquine. This could be due to the release of virions from intracellular vesicles [7], which were likely to be suppressed under chloroquine treatment. Chloroquine treatment at 40 µM effectively decreased viral load by at least three logs from as early as day 1 post-infection. This suppressed viral replication was maintained throughout the entire infection follow-up (Figure 1B).

#### 3.1.2. Pharmacokinetics of Chloroquine in Macaque

To confirm that plasma concentrations can reach the inhibitory concentration range shown in the in vitro studies (Figure 1A,B), three groups of two NHPs were given chloroquine either orally (7 and 14 mg/kg) or subcutaneously (14 mg/kg) for five consecutive days. On day 5 post-treatment evaluation of chloroquine uptake kinetics was done with Cmin that was obtained just before the 6th treatment (time 0 on Figure 1C). As shown, the route of treatment influenced plasma concentration kinetics (Figure 1C). Orally treated NHPs had a chloroquine plasma concentration below the expected inhibitory concentrations and large variation of curve evolution, whereas subcutaneous administration gave similar drug plasma concentrations in the two macaques, with a mean Cmax of 5.16 µM at 1 h post-treatment, a Cmin of 1.8 µM (after five days of treatment, dotted line, Figure 1C). These concentrations are very similar to those reported for malaria patients [23] and to the IC_50_ obtained for CHIKV-infected Vero E6 cells [12,13]. No hepatic toxicity was observed, with plasma levels of AST and ALT remaining in the normal range of 28–58 IU and 17–40 IU/mL, respectively. As such, chloroquine was subsequently delivered subcutaneously to the macaques for downstream studies.

#### 3.1.3. Chloroquine Treatment Exacerbates Acute Chikungunya Fever in Macaques

**Figure 2:**
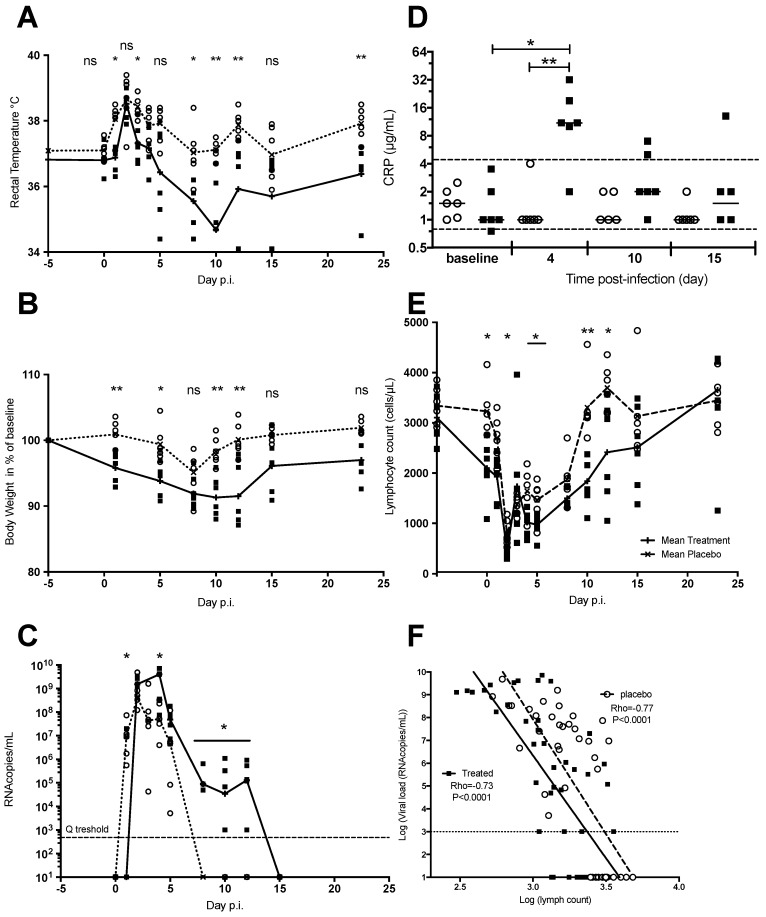
Exacerbation of acute chikungunya in macaques that were treated with chloroquine. Macaques randomly assigned to the placebo (*n* = 6, open circles, median shown as a dotted line) or chloroquine (*n* = 6, closed squares, median shown as a solid line) groups were treated for five days before infection with CHIKV. Treatment was then continued for 10 days. (**A**) Fever and hypothermia, as determined by measurements of rectal temperature; (**B**) Body weight loss in treated animals (percentage of baseline); (**C**) Plasma viral load, as measured by RT-qPCR; (**D**) CRP determination in plasma (* *p* < 0.05 Wilcoxon rank test). Global analysis by Kruskal-Wallis test, *p* = 0.0144 for the chloroquine group, not significant for the placebo group. The two dotted lines indicate the normal range of plasma CRP levels that were obtained from 15 healthy macaques; (**E**) Lymphopenia, as assessed by lymphocyte count kinetics; (**F**) Absolute lymphocyte counts are correlated with plasma viral load during acute chikungunya (Spearman’s rank correlation test). Mann & Whitney test when comparing placebo and treated animals; ns: not significant. * *p* < 0.05; ** *p* < 0.01. Wilcoxon rank test *p* values, for comparing the data at a given time point with baseline values (before treatment), are given in the text. Dotted horizontal line in C and F are the lower limit of quantitation by Q-RT-PCR.

To understand the effect of chloroquine on CHIKV infection in vivo, we mimicked pre-exposure prophylaxis by subcutaneous administration of chloroquine (14 mg/kg/day) or placebo to two groups of six NHP. Intravenous inoculation of 100 AID50 of CHIKV was performed 1 h after the 6th chloroquine treatment on day 5 (Figure 1D) [16]. Treatment was subsequently continued for 10 days after infection. Interestingly, a febrile episode occurred, peaking at two days-post-infection (dpi), in both placebo- and chloroquine-treated NHPs (*p* < 0.05 Wilcoxon peak versus baseline for each group, respectively) (Figure 2A). Whereas, body temperature in the placebo-treated animals returned to normal by 7 dpi, significant hypothermia was experienced by several chloroquine-treated NHP, between 5 to 10 dpi post-infection (*p* = 0.031 M&W test between the two group at this date), suggesting a more severe disease (Figure 2A). In addition, chloroquine-treatment also resulted in significant weight loss (e.g., five days after treatment initiation and before CHIKV infection) (*p* = 0.0043 M&W) that remained significant at 12 dpi (two days after the end of chloroquine treatment, *p* = 0.031 M&W).

NHPs in the placebo-treated group displayed a slight drop in body weight upon CHIKV infection, which recovered starting 8 dpi (Figure 2B). This suggests that the chloroquine treatment was the principal cause of the severe weight loss in treated NHPs, probably through a synergistic effect with infection.

Plasma viral load was detected as early as 1 dpi, peaked at 2 dpi (1.17 ± 0.74 × 10^9^ vRNA/mL) in the placebo-treated group before declining to become undetectable at 8 dpi (Figure 2C). On the other hand, the plasma viral load was significantly lower at 1 dpi in chloroquine-treated NHPs (*p* = 0.0379 M&W), before rebounding to peak between 2 and 4 dpi, obtaining values (peak value of 3.34 ± 1.10 × 10^9^ vRNA/mL) that were significantly higher than that in the control group (*p* = 0.045 M&W). Moreover, chloroquine-treated animals still had detectable viral loads of 10^3^–9.2 × 10^5^ vRNA/mL at 8 to 12 dpi (Fisher’s exact test, *p* < 0.001; Figure 2C), indicating that treatment delays virus clearance. 

Consistent with the observed exacerbation of acute CHIKV infection, serum C-reactive protein (CRP) concentration was high only in the chloroquine-treated group at 4 dpi (*p* < 0.05 M&W), possibly indicating the presence of a stronger infection-related inflammation (Figure 2D), despite the reported anti-inflammatory properties of chloroquine (for review, Solomon and Lee [24]). As previously reported, acute CHIKV infection in NHPs is associated with transient lymphocytopenia [16]. Prior treatment with chloroquine for five days significantly decreased the absolute lymphocyte counts from 3104 ± 199 to 2098 ± 235 cells/µL as compared to the placebo group (*p* < 0.032 Wilcoxon) (Figure 2E). However, the slope of correlation (Spearman *p* < 0.0001) with plasma viral load was similar between the two groups (*p* = 0.5304 Fisher’s exact test, Figure 2F). Nevertheless, lymphocytopenia was subsequently more prominent in the chloroquine-treated group when compared to the placebo group, with nadirs of 459 ± 64 and 795 ± 105 cells/µL, respectively, detected at 2 dpi (Figure 2E, *p* = 0.026 M&W). Significant differences between the groups remained until 12 dpi (*p* < 0.05 ANOVA+ Dunnet post-test), indicating that recovery was much slower in the chloroquine-treated NHPs, and this could possibly have related to the greater viremia observed in these animals.

#### 3.1.4. Chloroquine Skews the Immune Response to CHIKV

The striking contrast between the in vitro antiviral activities of chloroquine and its exacerbation of acute chikungunya disease in vivo in NHPs suggests that chloroquine could display other effects when given in vivo. To further understand the effect of chloroquine on CHIKV infection, we assessed the various markers of the innate and adaptive antiviral immune response in these placebo- and chloroquine-treated NHPs.

**Figure 3:**
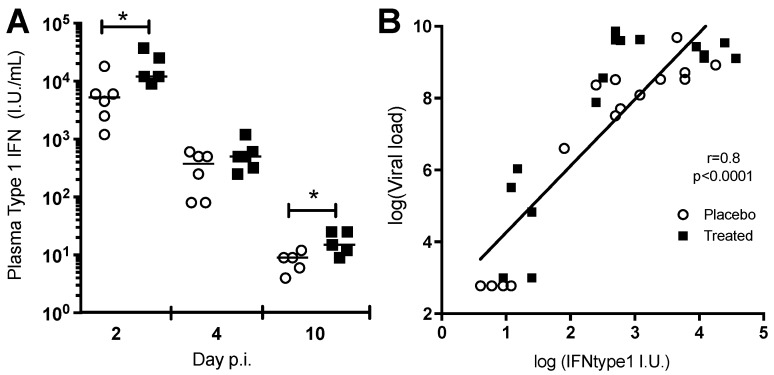
The type 1 interferon response and plasma viral load during acute chikungunya. (**A**) Type 1 interferon concentration was higher in the chloroquine group (black squares) than in the placebo group (open circles) on day 2 pi (viral load peak) and day 10 pi (during clearance). * *p* < 0.05, Mann & Whitney test; (**B**) Type 1 interferon concentration is correlated with plasma viral load during acute chikungunya (Spearman’s rank test, rho = 0.8, *p* < 0.0001).

Type I interferon (IFN-I) response is a very early, critical step in the innate immune response to acute viral infection. We used a biological assay to determine serum IFN-I concentration in macaques at 2, 4, and 10 dpi. Chloroquine treatment led to higher serum IFN-I concentrations than did placebo treatment, at both 2 and 10 dpi. (Figure 3A). IFN-I concentration was significantly correlated with viremia in both groups, suggesting that the increase in IFN-I concentration resulted from an increase in viremia, rather than a direct effect of chloroquine (Figure 3B; Spearman rho = 0.8, *p* < 0.0001).

**Figure 4:**
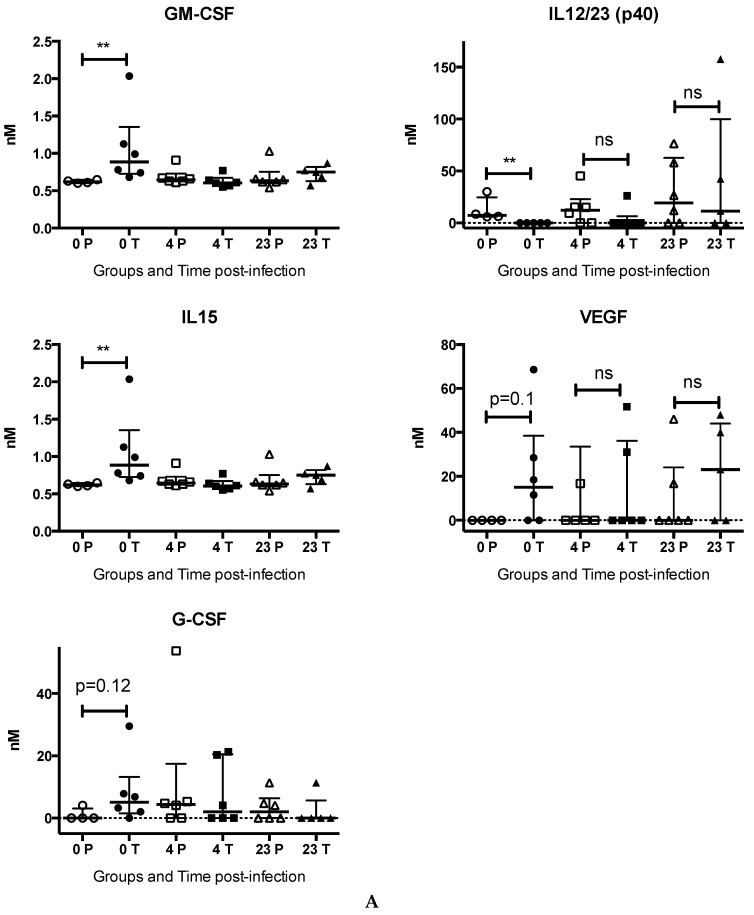

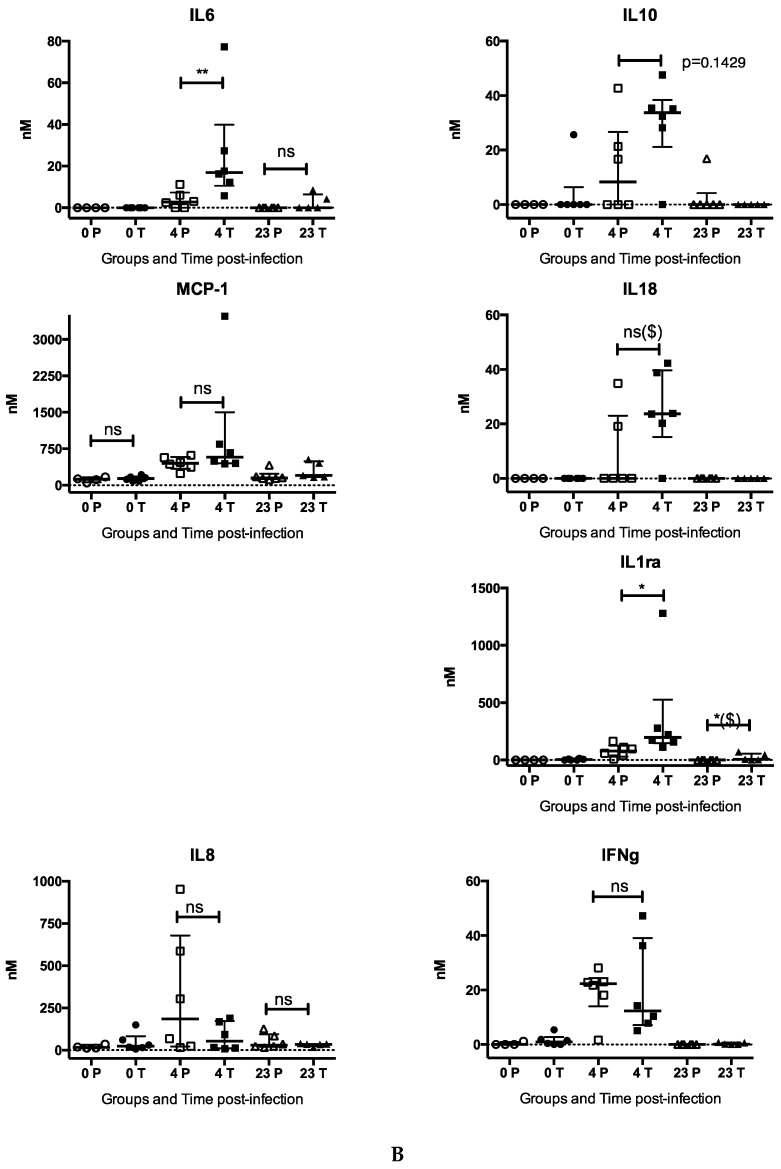
Evaluation of plasma inflammatory mediators in chloroquine treated (T, black symbols) versus non-treated (Placebo, P, open symbols) animals before infection (0P, 0T), and at 4, 23 days post-infection. Macaques were treated with chloroquine five days, and then were inoculated with 100 AID50 of CHIKV (day 0). Plasma grow-factor, cytokines and chemokines induced by the CHIKV infection were assayed using Luminex assays at day 0 before infection, four days post infection, and at day 23 post-infection thus 13 days after resolution of chloroquine treatment (see material and methods). (**A**) Chloroquine treatment induced variation in the expressed cytokines and chemokines: GM-CSF, IL-15, G-CSF, IL-12/23 (p40), and VEGF. Kruskal-Wallis then M&W test, ** *p* < 0.01, ns: not significant; (**B**) Cytokines significantly increased after infection (all *p* < 0.001: IL-6, MCP-1, IL-8, IL-10, IL-18, IL-1RA, IFNγ; in chloroquine treated (dark symbols) versus non-treated (open symbols) animals. Comparison between groups: Kruskal-Wallis then M&W test, ** *p* < 0.01, * *p* < 0.05; $ *p* < 0.07, ns: not significant.

Cytokine profiling using Bioplex technologies was performed retrospectively on samples that were collected from both groups of macaques at different times—(time 0 post-infection (pi), after five days of treatment (dt), 4 dpi (thus 9 dt), and 23 dpi (13 days since the end of treatment, a time where chloroquine was observed to be cleared from the system). Five days of chloroquine treatment alone induced an increase in GM-CSF, IL-15, VEGF, and G-CSF that is significant for GM-CSF and IL-15. In opposite, there was a significant decrease of IL12/23(p40) in these animals (Figure 4A), which probably signals the immune-modulation effect of chloroquine. The others tested cytokines, as MCP-1 and IL-8 or IL-6, IL-10, IL-18, IL-1RA and IFNγ normally undetectable in healthy animals, were not modified by the five days pre-treatment, but were significantly induced by the CHIKV infection (*p* < 0.05 Friedman test, Figure 4B). Interestingly, the increase is often less significant in non-treated animals. By contrast, in chloroquine treated animals, CHIKV infection induce a normalisation of the first group of growth factors and cytokines (Figure 4A) with a delay of IL-12(p40) and led to greater inflammation, with IL-6, MCP-1 being highly induced (Figure 4B). When comparing treated versus non-treated, IL-6, which was characterized by a very sharp peak at day one (data not shown see [16]), remained significantly higher in chloroquine treated animals, as with IL-1ra, while there was a non-statistically significant tendency (0.07 > *p* > 0.05) for an increase in MCP-1, IL-10, and IL-18 (Figure 4B).

Interestingly, the lower IL-8 and IFNγ responses in chloroquine treated animals (Figure 4B) might be related to the decrease of IL-12/23 induced by the treatment.

**Figure 5:**
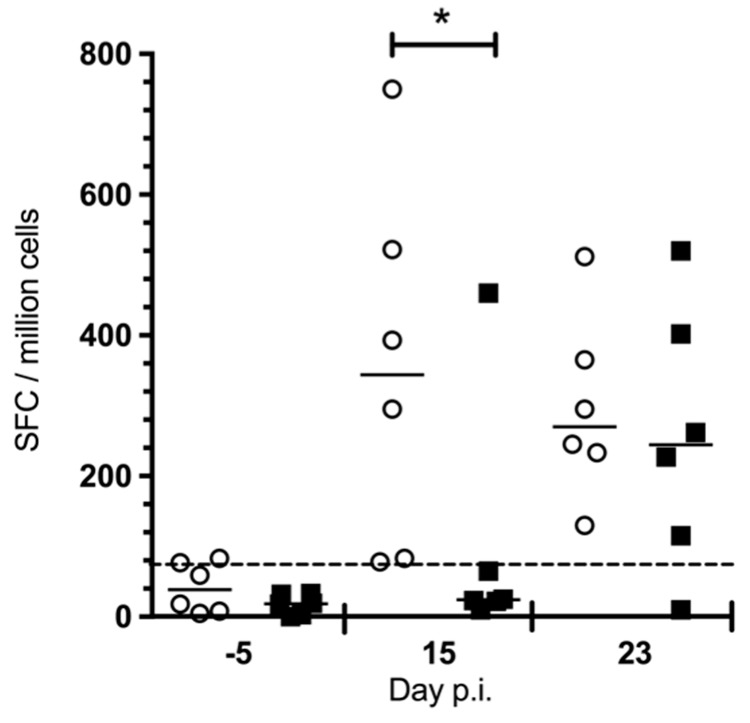
CHIKV-specific cell-mediated immunity is delayed by chloroquine treatment. The cell-mediated anti-CHIKV immune response was assessed by IFN-γ ELISPOT assays on leukocytes that were stimulated with CHIKV antigen in vitro, before treatment and infection (−5 p.i.), and on days 15 and 23 p.i. Chloroquine treated (black squares) versus non-treated (open circles). * *p* < 0.05, M&W test.

Adaptive immunity to acute CHIKV infection develops rapidly in both humans and animals [16,25,26,27]. We used IFN-γ ELISPOT assay as a robust indicator of virus-specific cell-mediated immunity. IFN-γ was detected in the placebo group at 15 dpi, whereas only one of the six treated macaques displayed a specific response at this time (Figure 5, *p* < 0.05 M&W).

This suggests that chloroquine may slow antigen-specific immune responses, thus causing a delay in cell-mediated adaptive immune response. Similarly, anti-CHIKV IgM titers were lower in the treated group than in the placebo group at 15 dpi (Chi^2^ test, *p* = 0.0432, Table 1). However, this difference was not significant at 23 dpi. Anti-CHIKV IgG titers were similar in both groups, but our data strongly suggest that chloroquine delays antigen-specific immunity, despite a robust IFN-I response.

### 3.2 Clinical Trial

Fifty-four patients with a biological confirmation of CHIKV infection were included in the CuraChik trial. Retrospective immunological analyses were possible for 46 of these (27 who received placebo and 19 who received chloroquine). Demographic, clinical, and biological characteristics at baseline relating to both placebo and chloroquine groups are presented in Supplemental Table S1.

At inclusion, all of the clinical and biological parameters were comparable between the two groups, except for the level of CRP that was higher in the chloroquine group (see Supplemental Table S1).

From inclusion (D1) to the end of the daily follow-up (D14), the patients from both chloroquine treated and placebo groups did not show any significant differences with respect to their daily clinical assessments (number of arthralgia and Visual Analogic Scale of health status, capacity to perform normal activity, and quality of sleep). Importantly, we used a GEE multivariate analysis to test the impact of a specific variable on the evolution of the disease (from D1 to D14). We found that chloroquine treatment did not modify the clinical evolution of the disease in terms of the number of arthralgia, Visual analogic scale of health status, the capacity to perform normal activity, or the sleep quality. However, being female and from older age group was associated with a higher level of arthralgia over time (D1 to D14). Telephone evaluation at D300, revealed that a larger fraction of patients treated with chloroquine experienced prolonged arthralgia when compared to those in the control group (Table 2).

#### Immunological Assay

**Figure 6:**
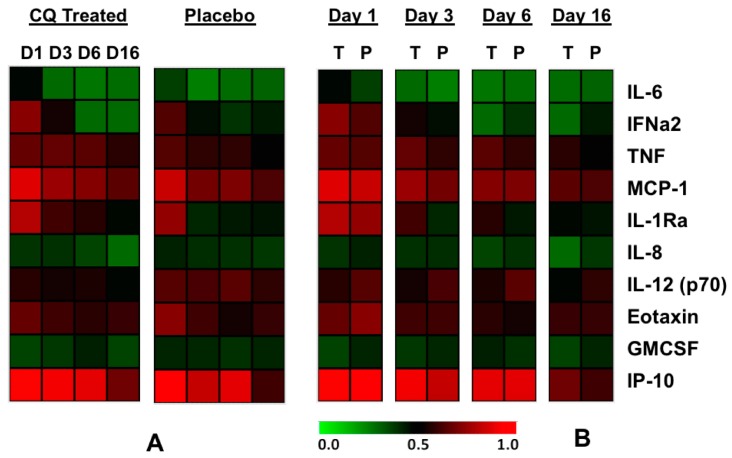
Heatmap of cytokines measured in each group. (**A**) General Comparison of chloroquine (CQ) treated vs Placebo treated groups (**B**) Comparing chloroquine treated (T) vs. Placebo treated (P) groups at each specific day. Heatplots are shown (top down) from the lower quantities (green) to higher quantities (red).

Cytokines (IL-6, IL-8, IL-1ra, IFNα, TNFα, and IL-12 (p70)), chemokines (MCP-1, IP-10, and Eotaxin), and growth factors (GM-CSF) were measured at four different time-points: day 1 (initiation), day 3 (midpoint), day 6 (endpoint) during chloroquine treatment, and day 16 (10 days after last chloroquine treatment). Modification of these soluble markers’ level have previously been reported to be associated with CHIKV infection in several human cohorts [28]. The expression levels of these analytes are plotted in a two-way cluster heat plot (Figure 6) and univariate analysis between placebo and chloroquine groups are presented in Supplemental Table S2.

At inclusion, despite randomisation according to plasmatic viral load, the levels of IFNα, IL-6, IL-8, and MCP-1 were significantly higher in the chloroquine group when compared to the placebo group (Supplemental Table S2). Altogether, these results imply that despite the randomization procedure at inclusion, the patients of the chloroquine group could have more severe initial disease, which is in accordance with the higher level of C-reactive Protein (CRP) that was found in the chloroquine group at baseline (see Supplemental Table S1).

Subsequently, to assess the impact of chloroquine treatment on the immunological response overtime in these patients, we used a GEE multivariate analysis approach after we have adjusted the values of the cytokines as a ratio on the first values. We thus found that chloroquine treatment was significantly associated with a faster decrease of Eotaxine, IL-6, and MCP-1 over time (*p* < 0.05, *p* < 0.001, and *p* < 0.05, respectively, see Annexe Supplemental Table S2, Figure 6B). At the chronic stage (D300 assessment) patients with persistent arthralgia had significantly higher level of TNFα at baseline, (*p* = 0.017) at D3 (*p* = 0.004) and at D16 (*p* = 0.011). In logistic regression multivariate analysis (using age, sexe, TNFα at baseline, and chloroquine treatment as independent variable), an increase of age, female gender, and chloroquine treatment were independently associated with persistent arthralgia at D300.

## 4. Discussion

African populations have made use of the anti-inflammatory properties of chloroquine for decades to cure febrile illnesses that were presumed to be malaria (personal observation). Chloroquine is also widely used against auto-immune diseases, like lupus or rheumatoid arthritis [29]. During the CHIKV outbreak in the Indian Ocean in 2005–2006, an increase in chloroquine use by the population of Reunion Island (an area free of malaria) was observed [30]. Experimental results in vitro suggested that chloroquine significantly inhibited CHIKV replication (consistent with previous data obtained with other alphaviruses) [12,31,32]. This raised important public health issues and led to a double-blind placebo-controlled randomized trial, that was conducted on Reunion Island and included adult patients with acute febrile arthralgia during the 2006 CHIKV epidemic. These patients received 600 mg of chloroquine per day for three days and then 300 mg for two days [12]. Here, we conducted a preventive trial carried out in NHPs, a well-established animal model for studying CHIKV pathogenesis [16], which was designed to assess whether the use of this drug could prevent CHIKV epidemic extension. The dose and administration route were determined on the basis of chloroquine pharmacokinetics in humans, to ensure that the data presented would be comparable and reproducible [23]. Neither trial reported a significant therapeutic effect. Moreover, the chosen regimen expected to be more efficient, according to the in vitro data (15 days of treatment initiated five days before infection) exacerbated acute chikungunya disease in the macaques.

This discrepancy between the efficacy of chloroquine in in vitro experiments involving Vero-E6 cells [12,13] and the results of clinical testing could be accounted for by the unfavorable balance of the antiviral and immunomodulatory effects of chloroquine in vivo. CHIKV induces IFN-β in fibroblasts, allowing for the intrinsic control of infection [33]. Conversely, chloroquine inhibits IFN-I responses in other paradigms [34] and these deleterious effects might have been missed in Vero-E6 cells, which do not produce IFN-I [35]. On the other hand, monocytes [36,37] and macrophages [16,22] are IFN-competent cells that are susceptible to CHIKV infection and are also critical to CHIKV pathogenesis. Given our observed antiviral effects of chloroquine in NHP-derived macrophages, the failure of chloroquine treatment in preventing and treating CHIKV infection in NHPs would reflect the undesired effects of chloroquine. Chloroquine exacerbates infection in other animal models by inducing a greater proinflammatory cytokine profile in Semliki forest virus (SFV) and encephalomyocarditis virus infected mice [38,39]. These two studies reported the effects on viral load similar to those describe here, however it was difficult to reconcile the observation with the known anti-inflammatory properties of chloroquine [40]. Chloroquine has known immune-regulating properties and has been used to treat rheumatoid arthritis and lupus erythematosus by reducing the inflammatory mediators that are present during the acute-phase response [40,41,42].

Whereas, the viremia in the placebo group was cleared by 5 dpi, CHIKV vRNA remained detectable in chloroquine-treated NHPs up to 12 dpi, two days after the end of treatment. IFN-I production was not impaired and was correlated with viral load, which is consistent with human data from Reunion Island [33]. Previous studies [33,43] have indicated a probable role of IFN-I in controlling viral replication. Nonetheless, despite a decrease of four to five orders of magnitude in virus levels between 5 and 7 dpi, chloroquine treatment in NHPs did not result in the completed clearance of CHIKV (Figure 2C). This indicates that the IFN-I-driven response is not capable of complete virus clearance in the treated macaques, a notion that was previously proposed by Werneke et al. [43]. This highlights the potential underlying immune-deficiencies that may prolong viremia during chloroquine treatment.

In the curative trial in human patients, chloroquine treatment did not modify the clinical and biological status, or the virus levels between days 1 and 3. Interestingly, despite a randomization procedure at baseline on CHIKV viral load, the immunological assays of the first sample revealed that patients that were included in the chloroquine group had a more severe disease (higher levels of IFNα, IL-6, IL-8, and MCP-1). This concordance with the higher levels of CRP observed in the chloroquine group at inclusion. To assess the potential impact of chloroquine administration in CHIKV treatment, we compared the profile of the immunological markers overtime between the two groups using thre GEE approach We found that chloroquine treatment was associated with a faster decrease of the level of Eotaxin, IL-6 and MCP1 over time. Furthermore, 300 days after treatment, advanced age, female gender and chloroquine treatment were independently associated with persistent arthralgia at D300. This may reflect the negative impact of chloroquine on the immunological response favoring a possible delayed of the CHIKV clearance, as it was shown in the NHP model.

Thus, the clearance of residual CHIKV depends on other antiviral responses that may be impaired by chloroquine treatment. We found that both CHIKV-specific humoral and cellular responses were delayed at 15 dpi. Subsequently, these responses recovered and were of similar levels to those that were observed in the placebo group at 23 dpi (13 days after the end of chloroquine treatment) (see Table 2 and Figure 5). Antigen-specific responses depend on the quality of antigen processing and presentation, which may be affected by chloroquine treatment [44]. Chloroquine inhibits TLR3 signalling, which is an important pathway in the response to viral infections [45], and antigen presentation on MHC class II molecules [44,46,47]. Possibly, this also impaired helper T cell activation throughout treatment, accounting for the hampered antigen-specific responses in chloroquine-treated macaques both from B cells (Table 1) and cytotoxic T cells (CTL, Figure 5). Conversely, chloroquine also promotes MHC class I presentation and CD8+ CTL responses [46,48], which would result in an immune boost situation rather than initial priming by CD4+ T cells. Thus, the initiation of continuous treatment for five days before CHIKV infection may have suppressed the anti-CHIKV response and concealed the anti-CHIKV activity of chloroquine. Viremia was detected one day later in chloroquine-treated macaques (2 versus 1 d.p.i. in the placebo group), suggesting that antiviral activity did occur in vivo, but it was likely to be outweighed by the immuno-suppressive effect of chloroquine. This highlights the importance of choosing the correct dose and administration schedule in achieving the best treatment outcome. For example, a single dose at peak fever, after antigen presentation to CD4+ helper lymphocytes has been initiated, might improve the recovery phase if it increases the CTL response [48]. The macaque model of CHIKV will be useful for testing this and other hypotheses.

## 5. Conclusions

The data from the two studies presented here, from a human clinical trial and an NHP trial, strongly indicate that chloroquine is not a suitable prophylactic or therapeutic option for CHIKV infection. Indeed, analyses of the data from the clinical trial suggests that the long-term residual effect of such a treatment might actually be deleterious with an increase in the chronic clinical manifestations in those that were treated with choloroquine.

## Figures and Tables

**Table 1:** CHIKV-specific antibody titers in infected macaque serum collected 15 days p.i.

Ig Class	Group	≤450	4000	37,000	≥110,000	*p* Value (Chi^2^ Test)
IgM titer	Placebo	0 ^$^	2	4	0	
	Chloroquine	1	5	0	0	0.0432
IgG titer	Placebo	0	0	4	2	
	Chloroquine	1	0	5	0	0.2111

^$^ The figures are the numbers of macaques with the given virus-specific Ig titer in each group.

**Table 2:** CHIKV-patient clinical status at Day 300 post treatment.

D300	Placebo Group *n* = 27	Chloroquine Group *n* = 19	*p*-Value
Recovery N/T ^$^ (%)	21/26 (80.8)	12/17 (70.6)	0.48 *
Presence of Arthralgia N/T (%)	6/26 (23.1)	8/15 (53.3)	0.08 *
N Joint involved (SD, mean-max)	1.5 (3.55, 0–12)	3.38 (4.6, 0–13)	0.038 **

Estimates of health status and ability to conduct everyday activities were reported by patients on daily questionnaires. Patients were asked for a self-assessment of these parameters on visual analogic scales (0 = very poor health status/capacity to perform everyday activities; 100 = normal health status/capacity to perform everyday activities). Here, are shown data at Day 300. Three patient were lost for follow up at day 300 (one in placebo, two in chloroquine), $: T = number of patient that answer to the evaluation, some patient do not answer to all of the questions. * Fisher’s exact test; ** Mann & Whitney test.

## Supplementary Materials

The following are available online at http://www.mdpi.com/1999-4915/10/5/268/s1, Figure S1: (A) Macaque primary fibroblast cell isolation procedure; (B) Differential impact of chloroquine or CHIKV on primary macaque fibroblast cells (passage 3 to 6). Table S1: Demographic characteristic, clinical and biological presentation at inclusion, Table S2: Univariate analysis of cytokine value in human groups.
