# Supplementary material for: Paradoxical Effect of Chloroquine Treatment in Enhancing Chikungunya Virus Infection
Source: Viruses. 2018 May 17;10(5):268. doi: 10.3390/v10050268 (PMC5977261; doi:10.3390/v10050268)
Supplement: Supplementary file 1 [file viruses-10-00268-s001.zip › suppl/4.viruses-302504 suppl 1.pdf]

Supplemental Table 1: Demographic characteristic, clinical and biological presentation at inclusion

INCLUSION	Placebo group	Chloroquine group	p-Value
**Age**			
Mean (SD, min-max)	38.9 (12.5, 18-66)	40.3 (12.8, 18-66)	0.76
**Gender N (%)**			
Male	21 (77.8)	10 (52.6)	0.11
Female	6 (22.2)	9 (47.4)	
**pre-existing orthopaedic illness**	2 (7.4)	0 (0)	0.7
**Number of Arthralgia**			
Mean (SD, min-max)	15.6 (10.8, 0-34)	16.1 (8.9, 2-30)	
**Intensity of Arthralgia N (%)**			
Absent to moderate	18 (66.7)	8 (42.1)	0.13
Important	9 (33.3)	11 (57.9)	
**Myalgia N (%)**			
Absent to moderate	25 (92.6)	14 (73.7)	0.11
Important	2 (7.4)	5 (26.3)	
**Health status**			
Mean (SD, min-max)	34.1 (18.3, 2-69)	27.6 (23.3, 0-75)	0.16
**Capacity to perform normal activity**			
Mean (SD, min-max)	34.4 (23.4, 0-94)	36.5 (27.6, 3-100)	0.8
**Quality of sleep**			
Mean (SD, min-max)	27 (28.6, 0-97)	35.4 (33.1, 0-95)	0.5
**Neutrophilia (G/l)**			
Mean (SD, min-max)	3.1 (1.4, 1.1-6.3)	3.7 (1.5, 1.7-7.1)	0.2
**Lymphocytes (G/l)**			
Mean (SD, min-max)	0.85 (0.7, 0.4-4)	0.78 (0.48, 0.3-1.9)	0.61
**Platelets (G/l)**			
Mean (SD, min-max)	168.2 (45.4, 106-294)	202.4 (67.8, 121-348)	0.078
**C-Reactive Protein (CRP) (mg/l)**			
Mean (SD, min-max)	40.5 (27.3, 9-113)	73.5 (56.3, 14-195)	**0.043**
**Viremia log10(copy/ml)**			
Mean (SD, min-max)	8.04 (1.04, 5.6-9.8)	8.5 (1.1, 5.6-10.1)	0.10
TOTAL	**27**	**19**	

Supplemental Table 2 : Univariate analysis of cytokines at inclusion (D1), day 3 (D3), day 6 (D6) and day 16 (D16) between placebo and chloroquine groups. Multivariate analysis of the cytokines evolution over time using GEE approach. CQ = chloroquine treatment from CuraChik clinical trial.

		**UNIVARIATE ANALYSIS**			**GEE analysis**	
		**Placebo group**	**Chloroquine group**	**p-Value**	**Independent Variable**	**p-Value**
		Mean (SD, min-max)	Mean (SD, min-max)			
**Eotaxin**	D1	181.7 (85.2 .61-383.7)	146.8 (78.5 .37.8-354.8)	0.09	Age	0.66
	D3	108.7 (38.5 .41-212.4)	109.4 (48 .50.5-234.5)	0.87	Sexe	**0.014**
	D6	87.2 (38.2 .35.4-175.6)	108.2 (83.4 .39.9-394.4)	0.54	Viremia D1	0.83
	D16	128.4 (86.2 .13-475.9)	114 (72.9 .37.8-326.6)	0.34	CQ	0**.044**
**GMCSF**	D1	10 (22.6 .0-118.5)	4.3 (4.3 .0-17.8)	0.22	Age	0.35
	D3	5.6 (11.9 .0-59.3)	5.2 (6.5 .0-28.2)	0.18	Sexe	0.26
	D6	8.6 (14.6 .0-50.3)	7.2 (12.7 .0-58.4)	0.33	Viremia D1	0.39
	D16	4.1 (6.3 .0-30.6)	4.2 (6 .0-27.5)	0.84	CQ	0.37
**IFNa2**	D1	68.9 (38 .9.9-171.3)	152.5 (139.7 .13.4-534.4)	**0.04**	Age	0.55
	D3	18.1 (21 .0-84.6)	33.9 (19.9 .0-66.2)	**0.004**	Sexe	0.075
	D6	13.1 (24.6 .0-100.5)	7 (8.5 .0-35.2)	0.41	Viremia D1	0.87
	D16	12.4 (25.8 .0-122.6)	4.7 (7.6 .0-30.4)	0.70	CQ	0.50
**IL12**	D1	22.2 (22.5 .0-96.6)	23.4 (34.1 .0-132.6)	0.54	Age	0.15
	D3	15.3 (7.2 .0-30.7)	21 (38.2 .0-129.2)	0.07	Sexe	0.24
	D6	16.7 (6.1 .8.7-29.4)	30.7 (71.5 .0-239.9)	**0.006**	Viremia D1	0.25
	D16	10 (10.2 .0-36.2)	19 (47.8 .0-205.5)	0.60	CQ	0.68
**IL1RA**	D1	250.7 (153.8 .40.3-666.6)	375.1 (284.3 .118.1-1141)	0.11	Age	0.89
	D3	45.1 (62.1 .0-291.3)	117.4 (82.1 .9.7-318.6)	**<0.001**	Sexe	0.051
	D6	43.6 (42.8 .0-190.6)	89 (83.2 .6-386)	**0.005**	Viremia D1	0.35
	D16	31.5 (47.3 .0-243.3)	72.4 (114.6 .0-517.7)	**0.049**	CQ	0.54
**IL6**	D1	13.7 (14.5 .2.3-66.8)	38.7 (39.1 .4.8-158.2)	**0.001**	Age	0.16
	D3	4.9 (8.1 .0-36)	6.3 (9.5 .0-32.2)	0.92	Sexe	0.30
	D6	4.2 (4.4 .0-13.6)	6.2 (10.7 .0-43)	0.98	Viremia D1	0.29
	D16	3 (5.8 .0-27.4)	3.7 (10 .0-41.9)	0.20	CQ	**<0.001**
**IL8**	D1	42.3 (28.7 .15.9-149.4)	24.7 (14.5 .3.9-61.6)	**0.005**	Age	0.55
	D3	30.5 (20.1 .6.1-91.4)	27.1 (14.6 .3.7-63.1)	0.99	Sexe	0.099
	D6	32.1 (35.9 .6.4-179.1)	20.6 (16.4 .1.8-65.5)	0.12	Viremia D1	**0.031**
	D16	18.3 (13.7 .0.8-53.4)	11.1 (12.1 .0.3-56.2)	**0.02**	CQ	0.87
**IP10**	D1	10881 (4267 .3866.4-19407.6)	9931.4 (3873.1 .2819.8-15234.6)	0.56	Age	0.29
	D3	7403.6 (3769.1 .626.8-16440.2)	9392.9 (4085.1 .3406.7-14078.4)	0.14	Sexe	0.40
	D6	6918.5 (3020.9 .1855.2-14534.5)	7203.8 (3783.6 .2916.9-13953.8)	0.98	Viremia D1	0.11
	D16	1439.2 (2041.7 .150.6-9212)	1075.3 (689.9 .311.8-2929.2)	0.58	CQ	0.68
**MCP1**	D1	2801.4 (1577.4 .579.1-6499.6)	4443.7 (2760.7 .1103.3-9613.3)	**0.039**	Age	0.96
	D3	963.4 (481.5 .219.6-2155)	1429.3 (1050.6 .425.4-5192)	0.059	Sexe	0.26
	D6	898.3 (661.9 .235.8-3624.8)	963.3 (581.7 .289.4-2973.9)	0.49	Viremia D1	0.98
	D16	519.4 (203.9 .262.6-1168.3)	506.9 (242.5 .243.8-1198.6)	0.61	CQ	**0.013**

**TNFa**	D1	23.4 (10.3 .7.1-52.4)	26.6 (10.2 .10.7-50.8)	0.17	Age	0.20
	D3	16.6 (5.7 .6.6-30.7)	27 (13.7 .10.1-68.1)	**0.003**	Sexe	0.57
	D6	16.7 (6.1 .8.7-29.4)	28 (26.5 .8.3-130.3)	**0.03**	Viremia D1	0.76
	D16	11.4 (5.4 .0-24.1)	16.2 (7.2 .7.6-39.6)	**0.02**	CQ	0.15
