# Supplementary material for: Paradoxical Effect of Chloroquine Treatment in Enhancing Chikungunya Virus Infection
Source: Viruses. 2018 May 17;10(5):268. doi: 10.3390/v10050268 (PMC5977261; doi:10.3390/v10050268)
Supplement: Supplementary file 1 [file viruses-10-00268-s001.zip › suppl/4.viruses-302504 suppl 2.pdf]

Supplementary Figure S1A : Macaque primary fibroblast cell isolation procedure

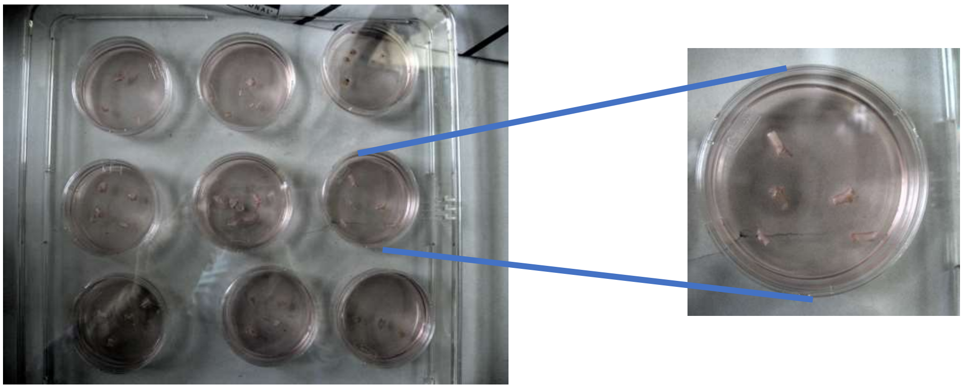

Piece of tendon obtained at necropsy from a healthy cynomolgus macaque was seeded in a small Petri dishes as described in Material and method.

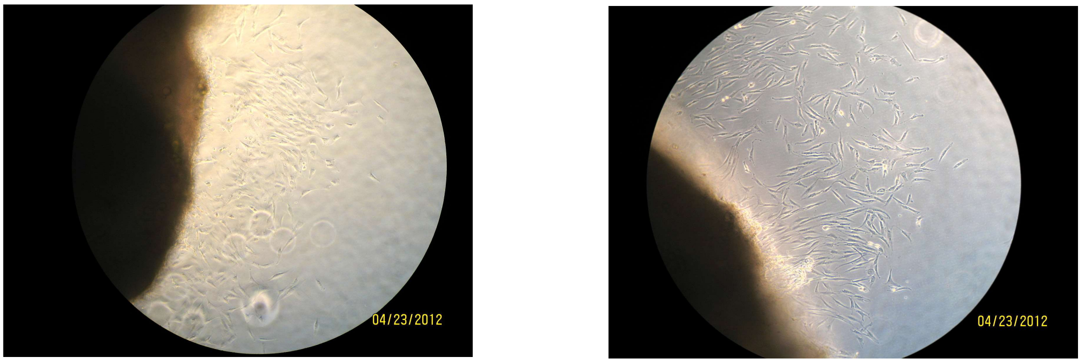

Two views of fibroblast cells migrating from the tendon explant at day 7 (magnification X10).

Supplementary Figure S1B : Differential impact of chloroquine or CHIKV on primary macaque fibroblast cells (passage 3 to 6). Picture are representative of 2 experiments performed in triplicate described in Figure 1B. Picture (all magnification 40X) were obtained at 48 hours post exposition **Panel A:** treated with 5 µM of Chloroquine and mock-infected; **Panel B:** treated with 40 µM of Chloroquine and mock-infected **Panel C:** treated with 5 µM of Chloroquine and CHIKV infected.

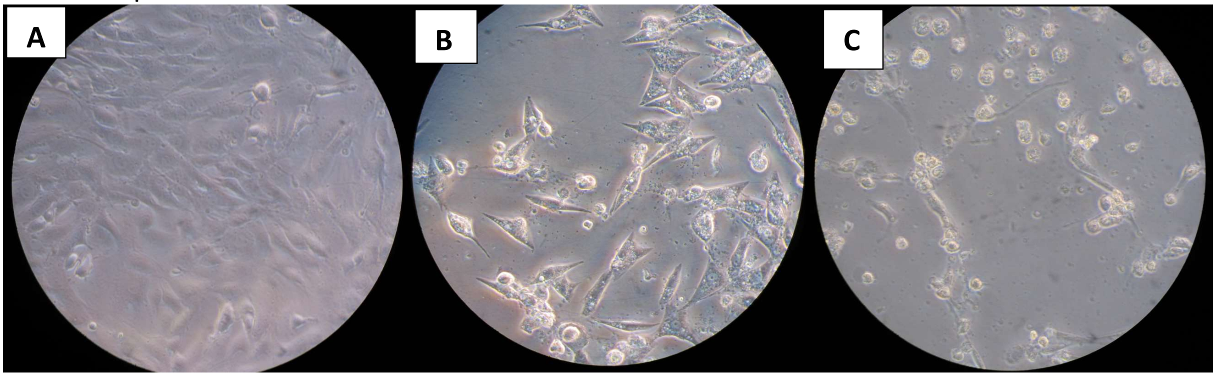

In panel A, normal aspect of fibroblasts stacked on the plate; in panel B, fibroblast were highly refringent and some of them evolved to round form with large cytoplasmic lysosomal structures; in panel C, close to all the cells are killed and detached from the plate.
